# High-Velocity Drag Racing Collision Leading to Rare Superior Mesenteric Branch Injury: A Case Report

**DOI:** 10.51894/001c.163745

**Published:** 2026-06-30

**Authors:** Christine Evans, Sabrina Miller, Zechariah Jean, Mitchell Ross, Kurt Erhardt, Micheal Buggia

**Affiliations:** 1 College of Osteopathic Medicine Michigan State University, East Lansing MI, USA; 2 Emergency Medicine Henry Ford Health System; 3 Emergency Medicine Henry Ford Hospital

**Keywords:** superior mesenteric artery branch injury, blunt abdominal trauma, motor vehicle accident, mesenteric ischemia.

## Abstract

**INTRODUCTION:**

Superior mesenteric artery (SMA) injury is a life-threatening condition that requires prompt identification and surgical intervention. The majority of SMA injuries occur secondary to penetrating trauma, with blunt trauma representing a rare etiology.

**CASE PRESENTATION:**

We report the case of a 20-year-old Asian male presenting to the emergency department (ED) after he sustained blunt trauma to the abdomen during a motor vehicle accident, causing a mesenteric defect and SMA branch rupture. On arrival to the ED, the patient was hemodynamically stable. A focused assessment with sonography for trauma (FAST) demonstrated hemoperitoneum, and computed tomography angiography (CTA) revealed active contrast extravasation from a branch of the distal jejunal branch of the superior mesenteric artery with an associated mesenteric defect. Shortly after imaging, the patient developed hemodynamic instability, requiring transfusion of blood products and an urgent exploratory laparotomy with ligation of the injured SMA branch and repair of the mesenteric defect. He was subsequently admitted to the intensive care unit (ICU) for postoperative management.

**CLINICAL DISCUSSION:**

This case highlights the diagnostic challenge of blunt SMA branch injuries, which may initially present with hemodynamic stability, but rapidly progress to life-threatening hemorrhage. A high index of suspicion should be maintained and patients should receive early imaging, close monitoring, and timely operative intervention are critical to prevent morbidity and mortality.

**CONCLUSIONS:**

Blunt trauma associated SMA branch rupture is an uncommon but potentially fatal injury. Clinicians should maintain a high index of suspicion for mesenteric vascular injury in patients with high-energy blunt abdominal trauma, even when initial hemodynamic parameters appear reassuring.

## INTRODUCTION

Injury to the superior mesenteric artery (SMA) is an uncommon but a highly lethal condition, most frequently resulting from penetrating trauma.^1^ Blunt abdominal trauma accounts for a small minority of the SMA injuries and presents a particular diagnostic challenge, as early clinical signs may be subtle or absent, even in the presence of significant vascular injury.^2,3^

Injury to the superior mesenteric artery (SMA) branches following blunt trauma is exceedingly rare, with only limited cases described in the literature.^2^ Delayed diagnosis of acute mesenteric vascular injury has been associated with bowel ischemia, sepsis, prolonged intensive care unit admission, and increased mortality. Traumatic injury to the superior mesenteric artery may precipitate acute mesenteric ischemia when perfusion is compromised.^3^ In such cases, diagnostic delay is associated with bowel necrosis, shock, and markedly increased perioperative and long-term mortality.^4,5^ Imaging findings may have subtle imaging findings further complicating timely recognition.^3^

This case is clinically relevant as it demonstrates how a patient with high-energy blunt abdominal trauma and initial hemodynamic stability can experience sudden decompensation due to occult SMA branch injury. It adds to the limited literature on blunt mesenteric vascular trauma by highlighting the diagnostic pitfalls, the importance of early computed tomography angiography, and the role of prompt operative management in preventing fatal outcomes.

## CASE PRESENTATION

A 20-year-old Asian male with no significant past medical history presented to the emergency department (ED) following a motor vehicle collision. He was the restrained driver of a vehicle traveling approximately 50 to 60 miles per hour that T-boned another vehicle during a drag race. Airbags deployed, and he sustained blunt abdominal trauma. The patient was able to self-extricate and ambulate at the scene. During transport to the ED, he remained hemodynamically stable.

On arrival, vital signs were as follows: 97.9 °F, heart rate was 89 beats per minute, respiratory rate of 18 breaths per minute, blood pressure of 108/78, and oxygen saturation of 97% on room air. He reported diffuse chest, abdominal, and bilateral hip pain. Physical examination was notable for a positive seatbelt sign with no focal neurologic deficits and dull movement of all extremities. A level II trauma activation was initiated. Extended Focused Assessment with Sonography in Trauma (E-FAST) exam was positive for free intraperitoneal fluid in Morison’s pouch, concerning hemoperitoneum.

**Figure 1.**
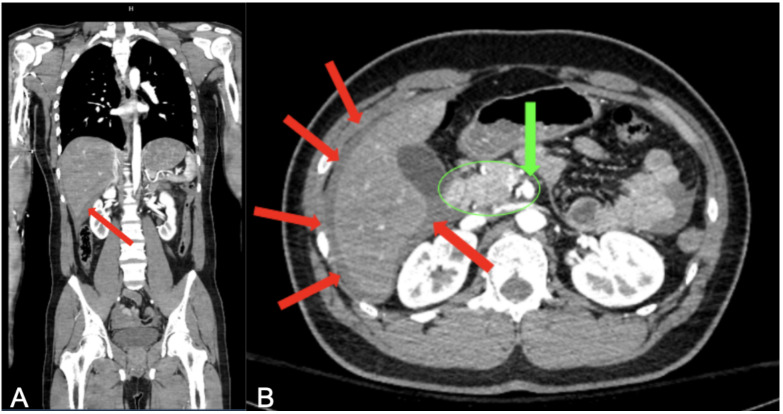
Computed tomography angiography demonstrating hemoperitoneum within the abdomen and pelvis. **Panel A (coronal view)** shows free intraperitoneal fluid (red arrows) and the superior mesenteric artery (green arrow) with active contrast extravasation (green circle). **Panel B (axial view)** demonstrates dependent free fluid consistent with findings appreciated on the FAST exam.

Given initial hemodynamic stability, computed tomography angiography (CTA) of the abdomen and pelvis was performed. Imaging demonstrated moderate hemoperitoneum with active contrast extravasation arising from a distal branch of the superior mesenteric artery (SMA), consistent with active arterial bleeding (Figure 1). Associated findings included contusion of the lower left anterior abdominal wall. Shortly after imaging, the patient developed hypotension, prompting transfusion of three units of packed red blood cells, one unit of fresh frozen plasma, and one unit of platelets. The patient was taken emergently to the operating room for exploratory laparotomy.

Intraoperatively, approximately two liters of intraperitoneal blood was evacuated. Active bleeding was identified from a ruptured distal jejunal branch of the superior mesenteric artery, which was ligated. A mesenteric defect was identified and repaired. A segment of ischemic small bowel was noted and resected, followed by primary anastomosis. Due to concern for ongoing edema and physiologic instability, the abdomen was left open with placement of a negative pressure temporary abdominal closure system. In the postoperative period, he demonstrated stable vital signs, including a blood pressure of 154/85 mmHg, oral temperature of 97.9°F, heart rate of 94 beats per minute, respiratory rate of 18 breaths per minute, and oxygen saturation of 100% while intubated. The patient was transferred to the Intensive Care Unit (ICU) postoperatively and empirically started on cefuroxime and metronidazole. He was sedated with fentanyl and midazolam and mechanically ventilated. Postoperatively, he developed lactic acidosis, which was managed with ventilator adjustments, including an increase in respiratory rate to 24 breaths per minute.

After two days in the ICU, the patient underwent a planned second-look laparotomy. The bowel appeared viable without evidence of ongoing ischemia. Minor adhesions were lysed via blunt dissection, and the abdomen was closed. Following successful extubation postoperatively, the patient’s vital signs were notable for a blood pressure of 164/70 mmHg, respiratory rate of 10 breaths per minute, heart rate of 99 beats per minute, and oral temperature of 99.5°F.

## RESULTS

**Figure 2.**
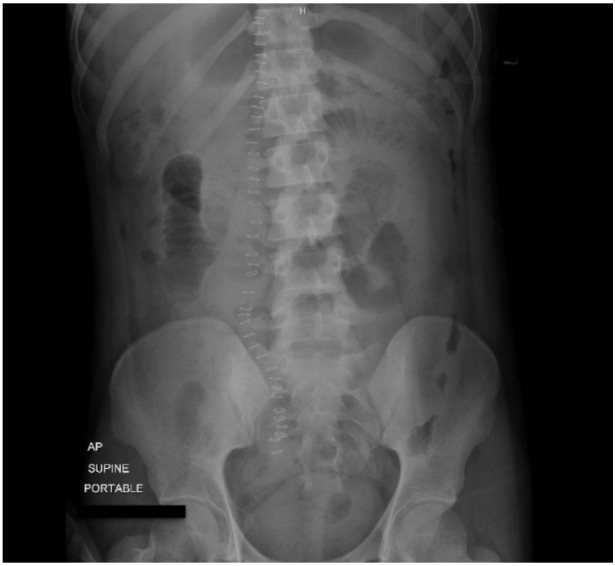
Abdominal radiograph demonstrating gas-filled small bowel loops consistent with post-operative ileus.

Postoperatively, the patient’s clinical course was complicated by anemia, post-operative ileus, sepsis, and pulmonary insufficiency. Anemia was attributed to intraoperative blood loss and postoperative hemodilution and was managed conservatively without additional transfusion. On postoperative day two following abdominal closure, abdominal radiography demonstrated mildly distended, gas-filled small bowel loops consistent with postoperative ileus (Figure 2), which was resolved with conservative management.

**Figure 3.**
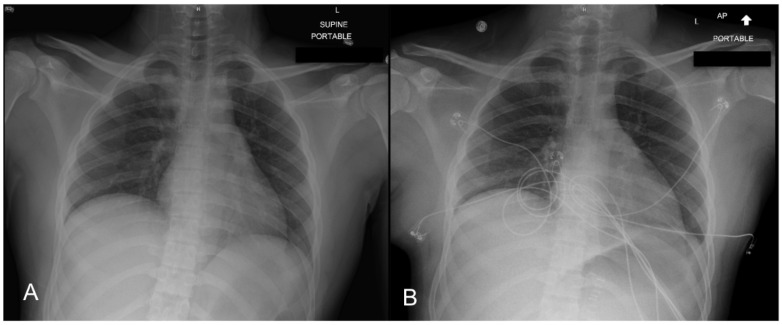
Comparison of chest radiographs: initial study (A) and hospital day four study demonstrating vascular congestion (B).

Chest radiography obtained on hospital day four demonstrated vascular congestion consistent with pulmonary insufficiency (Figure 3), which improved with supportive care. During hospitalization, the patient developed sepsis managed by a fever up to 101.2 °F.

Broad-spectrum antimicrobial therapy with ceftriaxone, metronidazole, and fluconazole was initiated, resulting in clinical improvement and resolution of fever. The patient demonstrated progressive functional recovery, with return of bowel function, advancement to oral intake, and stabilization of hemoglobin levels. He met discharge criteria and was discharged in stable condition.

The patient re-presented to the emergency department 10 days following discharge for evaluation of the surgical incision. The patient endorsed mild abdominal discomfort, which was adequately controlled with over-the-counter acetaminophen and ibuprofen. Physical examination revealed a linear abdominal incision with staples in situ, without evidence of surrounding erythema, warmth, drainage, dehiscence, or other signs of infection. The patient was advised to follow up with primary care and to attend an outpatient follow-up appointment with the operating surgeon the following week.

## CLINICAL DISCUSSION

Mesenteric injury occurs in approximately 1% to 5% of blunt trauma cases, with injury to the mesenteric vasculature representing the most frequently injured vessel in the abdomen.^3^ Although mesenteric vessels have been found to be the most commonly injured abdominal vessels in vascular trauma,^6^ true injury to the superior mesenteric artery (SMA) itself is distinctly uncommon, representing fewer than 1% of abdominal vascular injuries and carrying substantial morbidity and mortality.^2,7^ Although major abdominal vascular injuries most commonly involve the aorta, iliac arteries, and inferior vena cava, all of which are associated with substantial mortality,^8^ rupture of smaller SMA branches carries a high risk of rapid hemorrhage, with reported mortality rates for SMA Injuries as high as 54%.^1^ Compared with previously reported cases, this patient demonstrated an unusual injury pattern involving isolated SMA branch disruption following blunt trauma, initial hemodynamic stability, and delayed decompensation with survival after operative intervention.

The clinical presentation of blunt bowel and mesenteric vascular injury is often subtle and nonspecific, contributing to diagnostic delays. Abdominal pain and tenderness have limited predicted value, and their absence does not exclude significant injury, particularly in patients with distracting injuries or altered mental status.^7,9^ This diagnostic challenge is especially relevant in mesenteric vascular injuries, where early ischemia or hemorrhage may not initially produce peritoneal signs, necessitating close observation with serial examinations in at risk patients.^9^

The seatbelt sign is an important external marker of significant blunt abdominal trauma and is associated with bowel and mesenteric injury. Approximately 12% of patients presenting with a positive seatbelt sign sustain bowel injury, most commonly involving the small bowel, large bowel, and spleen.^3,9,10^ These injuries are thought to result from deceleration and compressive forces that generate shear stress at fixed mesenteric attachment points, predisposing to vascular stretch or avulsion.^11^ Even in alert patients without early abdominal symptoms, the presence of a seatbelt sign warrants definitive imaging and close monitoring.^11,12^ This mechanism likely contributed to the SMA branch injury observed in the present case.

In hemodynamically stable patients, contrast-enhanced computed tomography (CT) remains the diagnostic modality of choice for suspected mesenteric injury.^12^ Early CT findings suggestive of mesenteric vascular compromise include mesenteric hematoma, active contrast extravasation, focal mesenteric stranding, abrupt vessel cutoff or irregularity, bowel wall hypoenhancement, and unexplained hemoperitoneum.^12,13^ Diagnostic delays remain common and are associated with worse outcomes; studies have reported that delays exceeding 24 hours occur in approximately 31% of patients with blunt bowel or mesenteric injury and are associated with increased morbidity and mortality.^9^ This case highlights the need for a high index of suspicion and consideration of repeat imaging or early operative exploration when clinical deterioration occurs despite initially equivocal imaging.^9^

Management of SMA branch injuries requires timely operative intervention. Currently, management strategies are largely guided by individual case reports due to the rarity of SMA branch injuries and the absence of universal treatment guidelines.^2^ Reported approaches include hemorrhage control via ligation or repair, resection of nonviable bowel, and temporary abdominal closure with negative pressure therapy when necessary.^1,14^ Second-look laparotomy is often warranted to reassess bowel viability and prevent delayed ischemia, as was demonstrated in this case.^14^ The patient’s survival contrasts with many reported SMA injuries and the importance of early recognition, staged operative management, and postoperative monitoring.

This case demonstrates the potential for delayed hemodynamic collapse following a blunt abdominal trauma and highlights the importance of recognizing high-risk clinical markers and identifying early imaging features of mesenteric vascular compromise. Timely diagnosis and intervention remain critical to improving outcomes in these rare but life-threatening injuries.

### Limitations

This report is limited by its single-patient design, which restricts generalizability of the findings to broader trauma populations. The follow-up duration was limited, preventing long-term assessment of long-term vascular patency, bowel function, and delayed complications related to mesenteric ischemia. Additionally, imaging findings in mesenteric vascular injury may be variable and evolve over time, which can complicate early diagnosis. Subtle or nonspecific radiographic features may lead to diagnostic ambiguity, particularly in patients who initially present hemodynamically stable. Despite these limitations, this case provides valuable insight into the recognition and management of rare superior mesenteric artery branch injuries following blunt trauma.

## CONCLUSION

This case highlights the potential for rapid clinical deterioration in patients with blunt abdominal trauma who initially appear hemodynamically stable. High-speed mechanisms of injury should prompt heightened suspicion for mesenteric vascular injury, even when early computed tomography findings are subtle or equivocal. Early recognition, close clinical monitoring, and timely operative intervention are essential to reduce morbidity and mortality in these rare but potentially fatal injuries.
